# Integrated Metabolomics and Proteomics Analysis Reveals Plasma Lipid Metabolic Disturbance in Patients With Parkinson’s Disease

**DOI:** 10.3389/fnmol.2020.00080

**Published:** 2020-06-30

**Authors:** Ling Hu, Mei-Xue Dong, Yan-Ling Huang, Chang-Qi Lu, Qian Qian, Chun-Cheng Zhang, Xiao-Min Xu, Yang Liu, Guang-Hui Chen, You-Dong Wei

**Affiliations:** ^1^Department of Neurology, The First Affiliated Hospital of Chongqing Medical University, Chongqing, China; ^2^Department of Neurology, Renmin Hospital of Wuhan University, Hubei General Hospital, Wuhan, China; ^3^Department of Neurology, Chongqing University Central Hospital, Chongqing Emergency Medical Center, Chongqing, China; ^4^Department of Neurology, The People’s Hospital of Tongliang District, Chongqing, China; ^5^Department of Neurology, The Affiliated Hospital of Southwest Medical University, Luzhou, China

**Keywords:** Parkinson’s disease, metabolomics, proteomics, lipid, sphingolipid metabolism, apolipoprotein

## Abstract

Parkinson’s disease (PD) is a common neurodegenerative disease in the elderly with a pathogenesis that remains unclear. We aimed to explore its pathogenesis through plasma integrated metabolomics and proteomics analysis. The clinical data of consecutively recruited PD patients and healthy controls were assessed. Fasting plasma samples were obtained and analyzed using metabolomics and proteomics methods. After that, differentially expressed metabolites and proteins were identified for further bioinformatics analysis. No significant difference was found in the clinical data between these two groups. Eighty-three metabolites were differentially expressed in PD patients identified by metabolomics analysis. These metabolites were predominately lipid and lipid-like molecules (63%), among which 25% were sphingolipids. The sphingolipid metabolism pathway was enriched and tended to be activated in the following KEGG pathway analysis. According to the proteomics analysis, forty proteins were identified to be differentially expressed, seven of which were apolipoproteins. Furthermore, five of the six top ranking Gene Ontology terms from cellular components and eleven of the other fourteen Gene Ontology terms from biological processes were directly associated with lipid metabolism. In KEGG pathway analysis, the five enriched pathways were also significantly related with lipid metabolism (*p* < 0.05). Overall, Parkinson’s disease is associated with plasma lipid metabolic disturbance, including an activated sphingolipid metabolism and decreased apolipoproteins.

## Introduction

Parkinson’s disease (PD) is a common neurodegenerative disease and is expected to affect 14.2 million people worldwide by 2040 (Dorsey and Bloem, 2018). Its typical clinical features are motor symptoms, which result from accumulated Lewy bodies and the remarkable death of dopaminergic neurons in the substantia nigra pars compacta (SNpc). In addition, Parkinson’s disease is correlated with many non-motor symptoms, some of which even precede the motor symptoms by more than 10 years, including constipation, hyposmia, anxiety, depression, and rapid eye movement sleep behavior disorder (Emamzadeh and Surguchov, 2018). These non-motor symptoms are probably due to the disturbance of various neurotransmitters and multiple nerve systems in addition to the dopaminergic neurons in the SNpc (Lv and Ae, 2015). Most idiopathic PD patients are sporadic, and various environmental exposures have been identified as being correlated with the development of PD. Results of genome-wide association studies have also identified 90 independent mutations in more than 20 genes that increase risk factors for Parkinson’s disease (Blauwendraat et al., 2019). Therein, the strongest genetic risk factor is the Asn370Ser mutation of β-glucocerebrosidase, with an odds ratio greater than 5 (Sidransky et al., 2009). The interactions between environmental and genetic risk factors are still under investigation.

Drugs that increase intracerebral dopamine levels or directly stimulate dopamine receptors remain as the most common forms of treatment for PD patients (Connolly and Lang, 2014). These drugs are mainly for symptomatic treatment without neuroprotection or a so-called disease-modifying function. Researchers have found that mitochondrial oxidative stress and inflammation reaction play important roles in the apoptosis of dopaminergic neurons during the occurrence and development of Parkinson’s disease (Wei et al., 2018). However, drugs targeting oxidative stress have exhibited little therapeutic benefit (Trist et al., 2019). Research also indicates that Parkinson’s disease is related to the dysregulation of protein homeostasis, including intracellular and membrane protein trafficking, protein aggregation, and protein degradation by the ubiquitin-proteasome or lysosome-autophagy systems (Deng et al., 2018). Thus, the potential pathogenesis should be clarified and more effective disease-modifying treatments are urgently needed for PD patients.

Metabolomics is a comprehensive assessment of the total endogenous metabolites in a biological system, and proteomics can evaluate alterations at the protein level (Mostafa et al., 2016). These omics data can help researchers make many discoveries in the alteration of molecules and metabolic pathways following gene expression in PD patients. Plasma is an easily obtainable, non-invasive, and informative biofluid from patients, making it perfect to explore the pathogenesis of many neuropsychiatric disorders (Hu et al., 2016). Herein, we employed untargeted metabolomics and proteomics analysis to identify molecular changes in PD patients. Furthermore, an integrated bioinformatic analysis was performed to explore the probable pathogenesis of PD based on the above findings (Dong et al., 2018a).

## Materials and Methods

### Objects

Parkinson’s disease patients were consecutively recruited from April 2016 to February 2017 in the Department of Neurology, the First Affiliated Hospital of Chongqing Medical University, according to the criteria reported in a previous research (Dong et al., 2017b). The inclusion criteria was: (i) PD patients diagnosed using the European Federation of Neurological Societies and the International Parkinson Movement Disorder Society’s European Section (Berardelli et al., 2013), and (ii) patients only taking dopamine analogs or dopamine receptor agonists. The exclusion criteria was: (i) those with secondary Parkinson’s disease or Parkinson-plus syndrome; (ii) patients suffering from tumor, heart failure, chronic obstructive pulmonary disease, nephritis, infectious diseases, or any other severe chronic disease at the time of enrollment; and (iii) any history of stroke, brain surgery, head trauma, motor neuro disease, Alzheimer’s disease, or mental diseases.

A same amount of age- and sex-matched healthy controls (HCs) were included from the Department of Physical Examination. The controls were also without any history of illness in the central nervous system or suffering from any other severe disease. This study was approved by the ethics committee of the First Affiliated Hospital of Chongqing Medical University and performed in accordance with the Declaration of Helsinki. Informed consent was obtained from all individual participants included in the study. Clinical data, metabolomics analysis, and proteomics analysis were blindly assessed or performed (Dong et al., 2017a).

### Clinical Data

The clinical data of all included participants were collected. Fasting plasma samples were obtained with an EDTA-K2 tube in the morning, and then stored at -80°C until experimental analysis. All the clinical scales were assessed by experienced neurologists. Statistical analyses were performed using Statistic Package for the Social Sciences 22.0 (IBM, Armonk, NY, United States). Categorical data are exhibited as absolute numbers while continuous data are exhibited as mean ± standard error. All the clinical data were compared between PD and HC groups using Pearson *Chi*-squared tests or Fisher exact tests for categorical data and Mann–Whitney *U*-tests with Bonferroni *post hoc* tests for continuous data, as appropriate (Dong et al., 2016).

### Metabolomics Analysis

We adopted a Waters UPLC I-class system equipped with a binary solvent delivery manager (Waters Corporation, Milford, United States) to perform the untargeted liquid chromatography-mass spectrometry-based metabolomics (thirty-six objects per group); the detailed procedure was described in a previous research (Dong et al., 2018a). After that, data sets including m/z, peak retention time, and peak intensity of each ion were obtained. The m/z-peak retention time pairs were used to identify each ion based on Metlin^1^ and Human Metabolome Database (HMDB)^2^ while the peak intensity was deemed as the level of a metabolite. The data sets were further reduced by removing any peaks with a missing value in more than 60% of the total samples.

The positive and negative peak data were merged and multivariate statistical analyses were performed by the SIMCA-P 13.0 software package (Umetrics, Umeå, Sweden). The partial least squares-discriminant analysis (PLS-DA) model was constructed to show statistical differences and identify metabolites differentially expressed between PD patients and healthy controls, and this model was further validated by a permutation test with 200 iterations. Metabolites with variable influence on projection values (obtained from the PLS-DA model) of greater than 1.0, fold change values of greater than ±2, and *p*-values of less than 0.05 (Dong et al., 2018b) were identified to be differentially expressed. These differentially expressed metabolites were then classified by chemical taxonomy based on the HMDB database, and metabolic pathway analysis was further performed by MetaboAnalyst 4.0^3^ (Chong et al., 2018).

### Proteomics Analysis

The plasma of twenty-seven randomized healthy controls and thirty randomized PD patients were merged into three pooled samples for proteomics analysis. The details of the proteomics analysis have also been depicted previously (Dong et al., 2018a). Afterward, tandem mass spectrometry spectra were obtained and searched using the MASCOT engine 2.2 (Matrix Science, London, United Kingdom). The differentially expressed proteins were recognized by fold change values of greater than ±1.2 and *p*-values of less than 0.05. Gene ontology (GO) enrichment from cellular component, molecular function, and biological process, and Kyoto Encyclopedia of Genes and Genomes (KEGG) pathway enrichment analyses were performed by Fisher’s exact test, considering the whole quantified protein annotations as the background data set. The obtained *p*-values from enrichment analysis was further converted into *q*-value using Benjamini-Hochberg correction and a *q*-value less than 0.05 was considered significant (Hochberg and Benjamini, 1990).

## Results

### Results of the Metabolomics Analysis

Thirty-six PD patients and healthy controls were separately included in the metabolomics analysis. The clinical data of these participants are shown in Table 1. No significant differences were observed between these two groups, including the levels of hemoglobin A1C (HbA1C) and blood lipid, or the incidences of diabetes mellitus and hypercholesterolemia. The mean UPDRS score and Hoehn–Yahr score of these PD patients were 41.11 ± 3.72 and 2.26 ± 0.17, respectively.

**TABLE 1:** Clinical data of PD patients and healthy controls included in the untargeted liquid chromatography-mass spectrometry-based metabolomics analysis.

Variable (SEM/%)	HC (36)	PD (36)	*p*-value	Variable (SEM/%)	HC (36)	PD (36)	*p*-value
Age (year)	62.16 ± 1.73	64.03 ± 0.95	0.348	HbA1c (%)	6.17 ± 0.20	6.08 ± 0.14	0.689
Gender, Male (%)	18 (50%)	18 (50%)	1	TC (mmol/L)	4.42 ± 0.12	4.53 ± 0.13	0.516
Smoking history (%)	10 (27.8%)	9 (25.0%)	0.789	TG (mmol/L)	1.53 ± 0.09	1.44 ± 0.13	0.6
Alcohol consumption (%)	2 (5.6%)	2 (5.6%)	1	HDL-C (mmol/L)	1.30 ± 0.06	1.33 ± 0.06	0.794
Hypertension (%)	15 (46.9%)	17 (53.1%)	0.617	LDL-C (mmol/L)	2.87 ± 0.12	2.91 ± 0.11	0.816
Diabetes mellitus (%)	4 (12.5%)	8 (25.0%)	0.2	Apo-A1 (g/L)	1.37 ± 0.04	1.34 ± 0.04	0.658
Hypercholesterolemia (%)	12 (37.5%)	9 (28.1%)	0.424	Apo-B (g/L)	0.91 ± 0.04	0.91 ± 0.04	0.992
BMI (kg/m2)	24.21 ± 0.57	23.00 ± 0.64	0.169	Lpa (mg/L)	190.76 ± 42.32	279.53 ± 57.03	0.216
UPDRS score	/	41.11 ± 3.72	/	Hoehn-Yahr score	/	2.26 ± 0.17	/

*PD, Parkinson’s disease; SEM, standard error of the mean; HC, healthy control; HbA1c, hemoglobin A1c; TC, total cholesterol; TG, triglyceride; HDL-C, high-density lipoprotein cholesterol; LDL-C, low-density lipoprotein cholesterol; Apo-A1, apolipoprotein A1; Apo-B, apolipoprotein B; BMI, body mass index; Lpa, lipoprotein a; UPDRS, Unified Parkinson’s Disease Rating Scale.*

**FIGURE 1:**
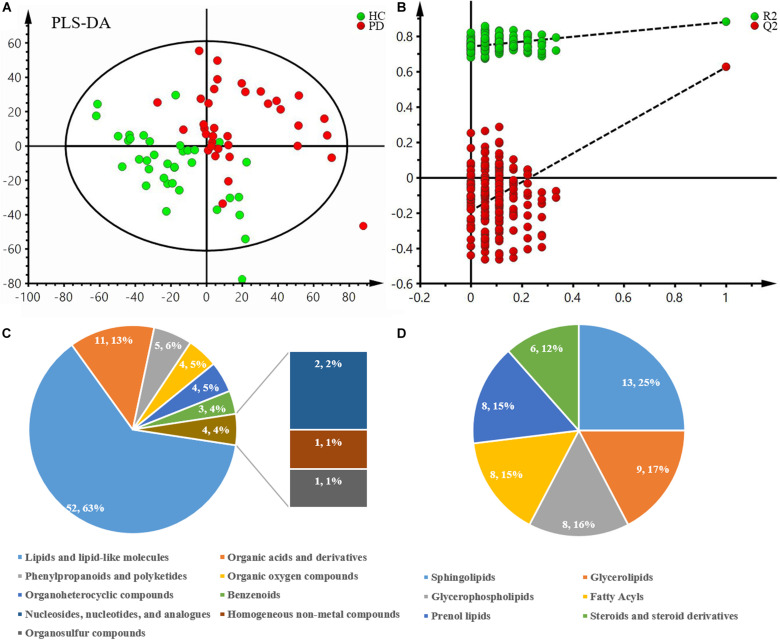
Multivariate statistical analysis of metabolomics and the classification of differentially expressed metabolites between PD patients and healthy controls. **(A)** PLS-DA score plot derived from liquid chromatography-mass spectrometry-based metabolomics analysis of PD patients and healthy controls. **(B)** Statistical validation of the PLS-DA model by permutation testing with 200 iterations. **(C)** Pie chart of the superclass of chemical taxonomy based on the annotations from Human Metabolome Database. **(D)** Pie chart of the further classification of lipids and lipid-like molecules. PD, Parkinson’s disease; HC, healthy control; PLS-DA, partial least squares-discriminant analysis.

After excluding internal standards, 6040 positive and 4363 negative peaks were detected in almost 98.81% of samples on average (Supplementary Table S1). Based on the above peaks, the PLS-DA score plot showed a significant difference between the PD and HC groups (R^2^X = 0.132, R^2^Y = 0.374, Q^2^ = 0.2) (Figure 1A). A permutation test with 200 iterations was performed and confirmed that the PLS-DA model was valid and not over-fitted because the original right R^2^ and Q^2^ values were observably higher than the corresponding permutated left values [*R*^2^ = (0.0, 0.746), *Q*^2^ = (0.0, -0.198)] (Figure 1B). According to the above results, eighty-three metabolites were differentially expressed between these two groups (Table 2). Of these, 63% were lipid and lipid-like molecules, 13% were organic acids and derivatives, 6% were phenylpropanoids and polyketides, 5% were organic oxygen compounds, 5% were organoheterocyclic compounds, 4% were benzenoids, 2% were nucleosides, nucleotides, and analogs, 1% was homogeneous non-metal compounds, and 1% was organosulfur compounds (Figure 1C). The lipid and lipid-like molecules were further categorized into sphingolipids (25%), glycerophospholipids (16%), glycerolipids (17%), fatty acyls (15%), prenol lipids (15%), and steroids and steroid derivatives (12%) (Figure 1D). These sphingolipids were ganglioside GD3 (d18:0/18:0), PS[15:0/20:4(8Z,11Z,14Z,17Z)], camellianin D, ceramide (d18:1/16:0), adrenorphin, ganglioside GD1a (d18:0/22:0), SM(d18:0/24:1(15Z)(OH)), thiamine(1+) diphosphate(1-), N-docosahexaenoyl phenylalanine, ganglioside GA2 (d18:1/12:0), SM(d18:0/22:0), glucosylceramide (d18:1/18:0), and AS 1–5.

**TABLE 2:** Key differentially expressed metabolites identified by untargeted liquid chromatography-mass spectrometry-based metabolomics analysis between PD patients and healthy control.

Compound Name	Compound ID^a^	m/z value	RT (min)	Ion mode	FC value^bc^	VIP value^c^	-log(*p-value*)^c^
Coenzyme A	HMDB0001423	750.0117	7.6840	Positive	0.4323	2.4685	5.292
Ganglioside GD3 (d18:0/18:0)	HMDB0011859	737.8448	2.9037	Positive	2.4947	2.1712	6.520
PS(15:0/20:4(8Z,11Z,14Z,17Z))	HMDB0012312	752.4899	7.6840	Positive	0.4153	2.5890	5.192
4-(Methylnitrosamino)-1-(3-pyridyl)-1-butanol	HMDB0030032	419.2401	3.8761	Positive	0.4434	2.0036	2.761
Lupulone	HMDB0030041	829.5719	2.8968	Positive	2.1749	2.4401	8.216
S-(Hydroxymethyl)glutathione	HMDB0004662	113.3748	8.8773	Positive	2.5248	1.5340	3.835
Glycinoprenol 11	HMDB0002003	737.7329	2.8968	Positive	2.4538	2.2782	7.317
TG(24:0/15:0/o-18:0)	HMDB47052	919.9253	2.9037	Positive	2.1137	1.8444	5.140
Camellianin D	HMDB0011920	620.4129	7.7665	Positive	0.4853	2.5231	5.238
Dipiperamide C	HMDB0034617	539.2666	1.9177	Positive	3.8676	1.1403	1.496
DG(24:0/24:0/0:0)	HMDB0007818	758.0465	9.2399	Positive	2.0050	1.0010	1.418
Iodide	HMDB0012238	65.4523	8.6504	Positive	2.4751	1.4282	1.689
TG[24:1(15Z)/20:5(5Z,8Z,11Z,14Z,17Z)/ 18:4(6Z,9Z,12Z,15Z)]	HMDB52343	947.7935	2.9037	Positive	2.2586	2.3058	7.459
PC[DiMe(11,3)/DiMe(13,5)]	HMDB0013456	922.6604	10.4327	Positive	2.2255	1.1533	1.568
Lucidenic acid M	HMDB0035973	925.5913	9.7743	Positive	3.0668	1.6801	3.860
Pangamic acid	HMDB0029949	900.5774	9.5886	Positive	2.1275	1.4546	3.466
Ceramide (d18:1/16:0)	HMDB0004949	520.5085	8.7673	Positive	2.6757	1.8323	4.813
Tyramine	HMDB0000306	69.5751	8.6504	Positive	2.1723	1.4084	1.849
Adrenorphin	HMDB0012081	948.5108	2.9037	Positive	2.5786	2.4955	8.273
Phenylacetyl-CoA	HMDB0006503	886.1336	9.9806	Positive	2.4328	1.2223	2.087
TG[22:1(13Z)/22:2(13Z,16Z)/o-18:0]	HMDB51764	947.9373	2.8968	Positive	2.1808	2.4274	8.070
Ganglioside GD1a (d18:0/22:0)	HMDB0011784	948.0813	2.9037	Positive	2.2465	2.4118	7.750
Kyotorphin	HMDB0005768	675.3525	8.1291	Positive	2.2765	1.4563	3.057
Kerdlan	HMDB0240277	853.6624	10.4396	Positive	2.0973	1.1777	1.769
Threoninyl-Glutamine	HMDB0029059	495.2411	1.8902	Positive	4.7886	1.1971	1.612
TG[18:4(6Z,9Z,12Z,15Z)/22:6(4Z,7Z,10Z,13Z,16Z,19Z)/ 18:4(6Z,9Z,12Z,15Z)]	HMDB0035640	883.6234	9.9875	Positive	2.5544	1.2463	2.114
SM[d18:0/24:1(15Z)(OH)]	HMDB0013469	829.6815	9.7674	Positive	2.5075	1.5106	3.333
3-Methylglutaconyl-CoA	HMDB0001057	858.0995	9.5886	Positive	2.4345	1.1968	2.040
Dynorphin A (6-8)	HMDB0012932	887.6266	9.9737	Positive	2.1618	1.3347	2.575
Thiamine(1+) Diphosphate(1−)	HMDB0011854	875.9981	6.9452	Positive	2.0855	1.1272	2.154
LysoPC[16:1(9Z)/0:0]	HMDB0010383	987.6122	9.7674	Positive	2.8140	1.4239	2.992
Dihydroceramide	HMDB0006752	686.6043	10.0268	Positive	2.6827	1.3579	2.580
D-Urobilinogen	HMDB0004158	591.3161	3.4380	Positive	2.4318	1.1614	1.396
N-Docosahexaenoyl phenylalanine	HMDB0006482	951.6668	10.4396	Positive	2.2063	1.3591	2.418
Alanyl-Asparagine	HMDB0028682	407.1886	1.8211	Positive	4.3023	1.2642	1.676
MG[0:0/22:4(7Z,10Z,13Z,16Z)/0:0]	HMDB0011554	840.6408	9.9875	Positive	2.7493	1.3265	2.430
Iopromide	HMDB0001493	814.1201	9.5886	Positive	2.3465	1.3392	2.809
Taraxacoside	HMDB0030055	797.2583	10.1989	Positive	2.3652	1.5763	3.144
Elaidic carnitine	HMDB0006464	851.7010	10.5890	Positive	2.0474	1.0989	1.502
10-Hydroxy-2,8-decadiene-4,6-diynoic acid	HMDB0031054	177.0544	1.6216	Positive	0.0001	2.2388	4.311
PE-NMe[18:3(6Z,9Z,12Z)/22:6(4Z,7Z,10Z,13Z,16Z,19Z)]	HMDB0009281	400.7789	9.4806	Positive	2.1062	1.7956	4.829
Mandelic acid	HMDB0000703	153.0546	1.6216	Positive	0.0078	2.3686	4.793
Sesaminol 2-O-triglucoside	HMDB0029556	831.2494	10.1989	Positive	2.2506	1.6307	3.334
Polyporusterone C	HMDB0000138	953.6222	10.1989	Positive	2.0235	1.5423	3.039
Ganglioside GA2 (d18:1/12:0)	HMDB0004888	991.6073	9.7537	Positive	2.0988	1.3269	2.273
Glucoconringiin	HMDB0001197	810.1524	9.9806	Positive	2.1748	1.4005	2.739
Mannosyl-(N-acetylglucosaminyl)2-diphosphodolichol	HMDB0012255	919.6318	9.7605	Positive	2.7027	1.6124	3.791
3-phenylprop-2-enoic acid	HMDB0000567	149.0597	1.6216	Positive	0.0047	2.3643	4.806
SM(d18:0/22:0)	HMDB0012091	789.6817	10.1989	Positive	2.3198	1.2248	2.279
Alpha-linolenyl carnitine	HMDB0006319	843.6455	9.9806	Positive	2.2240	1.4152	2.813
Perulactone B	HMDB0030119	977.6050	10.1989	Positive	2.8190	1.5222	2.913
PE[DiMe(11,3)/DiMe(13,5)]	HMDB0008682	880.5994	9.8293	Positive	2.0844	1.7057	4.208
Ebastine	HMDB0035995	939.6535	10.4327	Positive	2.2330	1.2181	1.702
TG[18:3(6Z,9Z,12Z)/18:4(6Z,9Z,12Z,15Z)/18:3(6Z,9Z,12Z)]	HMDB0010470	871.6774	10.4396	Positive	2.1182	1.2193	1.698
7-chloro-2-(3,4-dimethoxyphenyl)-3,5,6-trihydroxy-8-methoxy-4H-chromen-4-one	HMDB0001484	816.1138	9.5955	Positive	2.0290	1.2475	2.113
(3beta,5alpha,9alpha,22E,24R)-5,9-Epidioxy-3-hydroxyergosta-7,22-dien-6-one	HMDB0032666	885.6326	10.1989	Positive	2.1876	1.5669	3.119
Cholesterol sulfate	HMDB0000653	931.6238	10.2009	Negative	2.3774	1.5823	3.259
Methylimidazole acetaldehyde	HMDB0004181	371.1829	3.3773	Negative	0.4504	2.1379	2.501
Vanillactic acid	HMDB0000913	211.0608	1.8145	Negative	0.0027	2.4279	4.091
PC[14:1(9Z)/22:2(13Z,16Z)]	HMDB0007921	764.5535	10.0221	Negative	2.0590	1.3654	2.573
2-Octenedioic acid	HMDB0000341	343.1414	1.7113	Negative	0.0230	1.4765	2.370
1,2,3,4-Tetrahydro-beta-carboline	HMDB0012488	515.2876	3.2529	Negative	2.0170	1.1922	1.332
Methyldopa	HMDB0011754	210.0768	1.6219	Negative	0.0000	2.5799	4.767
PE[20:2(11Z,14Z)/24:1(15Z)]	HMDB0009311	834.6232	9.8316	Negative	2.1900	1.8322	5.080
Glucosylceramide (d18:1/18:0)	HMDB0004972	708.6159	10.0221	Negative	2.9864	1.3608	2.665
*cis-*Hydroxy Perhexiline	HMDB60644	585.5305	9.2354	Negative	2.7983	1.3734	2.780
TG[18:4(6Z,9Z,12Z,15Z)/20:5(5Z,8Z,11Z,14Z,17Z)/ 18:4(6Z,9Z,12Z,15Z)]	HMDB55513	873.6569	9.7697	Negative	2.8400	1.6794	4.108
Cinncassiol D1 glucoside	HMDB0034677	513.2730	3.3567	Negative	2.4747	1.6398	2.277
AS 1-5	HMDB0032843	714.5535	8.7898	Negative	2.1915	1.4575	2.933
Glycochenodeoxycholic acid 3-glucuronide	HMDB0002579	606.3294	3.1567	Negative	2.5622	1.6289	2.650
TG[14:1(9Z)/14:0/14:1(9Z)]	HMDB47724	717.5925	9.4073	Negative	2.1038	1.3365	2.913
Asparaginyl-Serine	HMDB0028740	437.1628	3.6093	Negative	0.4650	1.8095	1.691
1-non-adecanoyl-glycero-3-phosphate	HMDB62322	903.5960	9.7766	Negative	2.6135	1.6610	3.764
Hexahydro-6,7-dihydroxy-5-(hydroxymethyl)-3-(2-hydroxyphenyl)-2H-pyrano[2,3-d]oxazol-2-one	HMDB0029234	278.0648	1.6219	Negative	0.0001	2.5755	4.609
PE[DiMe(13,5)/MonoMe(13,5)]	HMDB61496	902.6103	9.8316	Negative	3.5042	1.6608	4.153
(2-hydroxy-2-{9-[(3-methylbut-2-enoyl)oxy]-2-oxo-2H,8H,9H-furo[2,3-h]chromen-8-yl}propoxy)sulfonic acid	HMDB0001511	421.0596	2.7668	Negative	0.4444	1.6591	1.564
(2S)-2-amino-3-[4-hydroxy-3-(sulfooxy)phenyl]-2-methylpropanoic acid	HMDB0142153	290.0342	1.6082	Negative	0.1225	2.1124	2.902
Asiaticoside	HMDB36656	979.5918	9.9829	Negative	2.2756	1.7261	4.518
Ponasteroside A	HMDB0034091	625.3600	4.2329	Negative	2.2531	1.0898	1.442
MG(12:0/0:0/0:0)	HMDB72863	821.6323	9.9829	negative	2.2034	1.6330	4.102
7-Chloro-6-demethylcepharadione B	HMDB0031833	340.0355	1.6219	Negative	0.0001	2.3257	3.726
Norpropoxyphene	HMDB0011627	974.5991	8.7760	Negative	2.3974	1.1448	1.437
Hordatine B glucoside	HMDB0030460	370.1782	4.0679	Negative	0.3956	1.7169	1.856

*^a^Compound ID was mainly exhibited based on the Human Metabolome Database (www.hmdb.ca). ^b^FC value was calculated as the ratio of the average mass response (area) between the two groups (FC value = PD/HC). Thus, FC values >1 indicate significantly higher levels in the PD group relative to the HC group while FC values <1 indicate significantly lower levels in the PD group. ^c^Only metabolites with FC values greater than ±2.0, VIP values greater than 1.0 and p-values less than 0.05 were deemed statistically significant. PD, Parkinson’s disease; RT, retention time; FC, fold change; VIP, variable influence on projection; HC, healthy control.*

**FIGURE 2:**
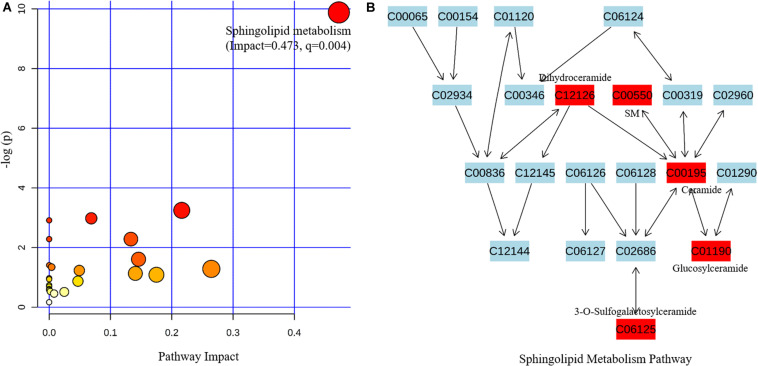
Metabolic pathway analysis based on the differentially expressed metabolites from the metabolomics data using MetaboAnalyst4.0. **(A)** Pathway analysis indicates sphingolipid metabolism is the only statistically enriched pathway. **(B)** In the sphingolipid metabolism pathway there are five differentially expressed metabolites involved, and all of them have increased in patients with Parkinson’s disease.

The related metabolic pathway is shown in Figure 2A, where only the sphingolipid metabolism pathway is significantly enriched (pathway impact = 0.473, *q*-value = 0.004). All six metabolites involved in sphingolipid metabolism had significantly increased, indicating that the pathway is activated in PD patients (Figure 2B).

### Results of the Proteomics Analysis

**FIGURE 3:**
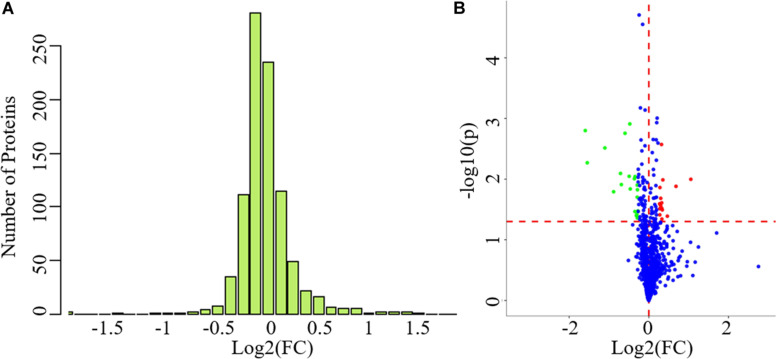
The exhibition of whole proteins detected in plasma and the differentially expressed proteins between PD patients and healthy controls. **(A)** Protein ratio distribution, **(B)** volcano plot showed FC values and *p*-values of all the identified proteins. Red dots represent increased differentially expressed proteins while green dots represent decreased proteins. FC value was calculated as the ratio of the average mass response area between the two groups (FC value = PD/HC). *p*-Value was calculated using Mann-Whitney *U*-test. PD, Parkinson’s disease; HC, healthy control; FC, fold change. Blue dots represent unchanged proteins. The horizonal red dashed lines indicate a *p*-value < 0.05 while the vertical red dashed lines indicate FC = 0.

The clinical data of all the participants included in the proteomics analysis are exhibited in Table 3. No statistical differences can be observed in these data, indicating that the following comparison of the two groups was reasonable. A total of 912 proteins were identified in plasma and protein ratio distributions between the two groups, which are shown in Figure 3A. Forty differentially expressed proteins were recognized, with fifteen proteins increased and twenty-five decreased (Figure 3B). The details of these proteins were shown in Table 4 and the seven differentially expressed apolipoproteins (apolipoprotein C-I, apolipoprotein C-III, protein APOC4-APOC2, apolipoprotein C-IV, apolipoprotein B variant, apolipoprotein B, and apolipoprotein M) were all significantly decreased.

**TABLE 3:** Clinical data of PD patients and healthy controls included in the tandem mass tag-based proteomics analysis.

Variable (SEM/%)	HC (27)	PD (30)	*p*-value	Variable (SEM/%)	HC (27)	PD (30)	*p*-value
Age (year)	66.59 ± 1.14	69.17 ± 1.50	0.184	HbA1c (%)	5.99 ± 0.18	6.17 ± 0.19	0.515
Gender, Male (%)	14 (51.9%)	19 (63.3%)	0.381	TC (mmol/L)	4.33 ± 0.17	4.32 ± 0.16	0.952
Smoking history (%)	9 (33.3%)	7 (23.3%)	0.402	TG (mmol/L)	1.49 ± 0.15	1.23 ± 0.14	0.206
Alcohol consumption (%)	3 (11.1%)	1 (3.3%)	0.53	HDL-C (mmol/L)	1.21 ± 0.08	1.24 ± 0.09	0.858
Hypertension (%)	15 (55.6%)	13 (43.3%)	0.357	LDL-C (mmol/L)	2.81 ± 0.15	2.73 ± 0.13	0.679
Diabetes mellitus (%)	2 (7.4%)	5 (16.7%)	0.51	Apo-A1 (g/L)	1.31 ± 0.05	1.30 ± 0.05	0.93
Hypercholesterolemia (%)	4 (14.8%)	6 (20.0%)	0.869	Apo-B (g/L)	0.89 ± 0.04	0.85 ± 0.04	0.403
BMI (kg/m2)	23.95 ± 0.69	22.79 ± 0.58	0.197	Lpa (mg/L)	201.19 ± 48.58	270.04 ± 66.49	0.407
UPDRS score	/	38.49 ± 3.98	/	Hoehn-Yahr score	/	2.40 ± 0.16	/

*PD, Parkinson’s disease; SEM, standard error of the mean; HC, healthy control; HbA1c, hemoglobin A1c; TC, total cholesterol; TG, triglyceride; HDL-C, high-density lipoprotein cholesterol; LDL-C, low-density lipoprotein cholesterol; Apo-A1, apolipoprotein A1; Apo-B, apolipoprotein B; BMI, body mass index; Lpa, lipoprotein a; UPDRS, Unified Parkinson’s Disease Rating Scale.*

**TABLE 4:** Key differentially expressed proteins identified by tandem mass tag-based proteomics analysis between PD patients and healthy controls.

Uniprot ID	Protein name (GeneName)	MW [kDa]	FC value^a^	*p*-value^b^
O75339	Cartilage intermediate layer protein 1 (CILP)	132.48	1.247	0.003
A9UFC0	Caspase 14 (CASP14)	27.65	2.077	0.010
Q5T619	Zinc finger protein 648 (ZNF648)	62.30	1.276	0.010
Q04756	Hepatocyte growth factor activator (HGFAC)	70.64	1.605	0.013
Q9UQ05	Potassium voltage-gated channel subfamily H member 4 (KCNH4)	111.62	1.213	0.014
E5RIF9	Carbonic anhydrase 1 (CA1)	16.31	1.230	0.021
P01023	Alpha-2-macroglobulin (A2M)	163.19	1.251	0.025
B7Z544	cDNA FLJ51742, highly similar to Inter-alpha-trypsin inhibitor heavy chain H4	98.29	1.206	0.026
Q6UWP8	Suprabasin (SBSN)	60.50	1.230	0.028
A0A087WYF1	Laminin subunit alpha-2 (LAMA2)	343.20	1.254	0.030
P04275	von Willebrand factor (VWF)	309.06	1.210	0.031
D3DQX7	Serum amyloid A protein (SAA1)	13.55	1.264	0.032
A0A125U0U7	MS-C1 heavy chain variable region	13.09	1.210	0.039
Q96K23	cDNA FLJ14838 fis, clone OVARC1001726, weakly similar to APICAL-LIKE PROTEIN	27.99	1.380	0.041
D6RF20	Vitamin D-binding protein(GC)	16.07	1.255	0.046
P02656	Apolipoprotein C-III (APOC3)	10.85	0.718	0.001
Q1W658	Follicle-stimulating hormone beta subunit (FSHB)	8.45	0.331	0.002
K7ER74	Protein APOC4-APOC2 (APOC4-APOC2)	20.04	0.661	0.002
Q04695	Keratin, type I cytoskeletal 17 (KRT17)	48.08	0.465	0.003
Q5NV68	V4-1 protein (V4-1)	11.23	0.342	0.005
Q59HB3	Apolipoprotein B variant	183.46	0.832	0.007
P04259	Keratin, type II cytoskeletal 6B (KRT6B)	60.03	0.611	0.008
K7ERI9	Apolipoprotein C-I (APOC1)	8.64	0.710	0.009
G5E968	Chromogranin A (CHGA)	34.25	0.786	0.009
Q99592	Zinc finger and BTB domain-containing protein 18 (ZBTB18)	58.32	0.830	0.009
P10720	Platelet factor 4 variant (PF4V1)	11.55	0.778	0.010
O95445	Apolipoprotein M (APOM)	21.24	0.831	0.011
A0A0G2JPR0	Complement C4-A (C4A)	192.75	0.620	0.012
Q99857	Tenascin-C	9.76	0.818	0.013
Q07507	Dermatopontin (DPT)	23.99	0.724	0.015
O95576	Pepsinogen	8.71	0.820	0.015
C0KRQ8	Glycoprotein hormones alpha chain (CGA)	10.20	0.542	0.016
P55056	Apolipoprotein C-IV (APOC4)	14.54	0.817	0.020
P35908	Keratin, type II cytoskeletal 2 epidermal (KRT2)	65.39	0.827	0.033
E1B4S7	Apolipoprotein B (APOB)	25.22	0.831	0.034
P80108	Phosphatidylinositol-glycan-specific phospholipase D (GPLD1)	92.28	0.785	0.034
H7C0N0	Inter-alpha-trypsin inhibitor heavy chain H1 (ITIH1)	51.83	0.811	0.039
H7BZ76	Latent-transforming growth factor beta-binding protein 1 (LTBP1)	28.80	0.806	0.040
Q7RTS7	Keratin, type II cytoskeletal 74 (KRT74)	57.83	0.807	0.040
B2RCB8	cDNA, FLJ95971, highly similar to Homo sapiens protocadherin 12 (PCDH12)	128.91	0.819	0.044

*^a^FC value was calculated as the ratio of the average mass response (area) between the two groups (FC value = PD/HC). Thus, FC values >1 indicate significantly higher levels in the PD group relative to the HC group while FC values <1 indicate significantly lower levels in the PD group. ^b^Only proteins with FC values greater than ± 1.2 and p-values less than 0.05 were deemed statistically significant. PD, Parkinson’s disease; MW, molecular weight; FC, fold change; HC, healthy control.*

**FIGURE 4:**
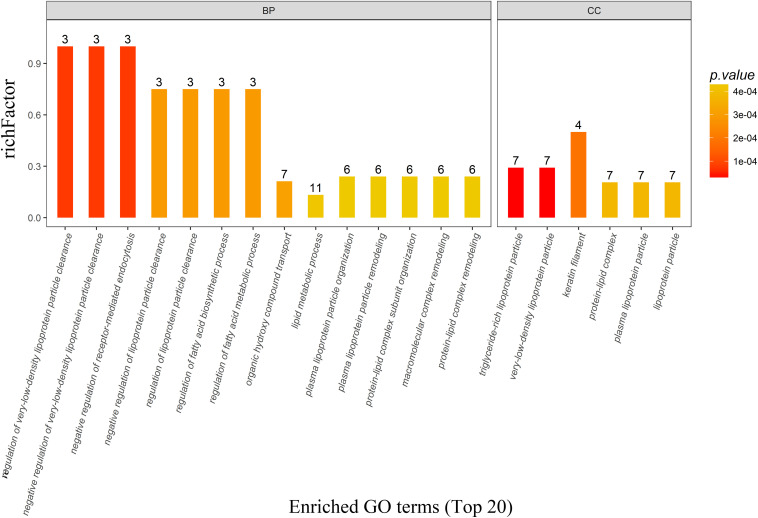
The top 20 most enriched GO terms based on proteomics analysis between PD patients and healthy controls. Five of the six top ranked GO terms from the cellular component and eleven of the 14 top ranked GO terms from the biological process were directly associated with lipid metabolism. BP, biological process; CC, cellular component; GO, gene ontology; PD, Parkinson’s disease.

There were 162, 25, and 22 GO terms significantly associated with biological processes, cellular components, and molecular function, respectively. The top 20 GO terms from GO enrichment analysis are shown in Figure 4 and Table 5. Five of the six top ranked GO terms from cellular components (very-low-density lipoprotein particle, triglyceride-rich lipoprotein particle, lipoprotein particle, plasma lipoprotein particle, and protein-lipid complex) were directly associated with lipids. The remaining top ranked GO term from cellular components was keratin filament. Eleven of the fourteen top ranked GO terms from biological processes were directly associated with lipid metabolism. These were regulation of very-low-density lipoprotein particle clearance, negative regulation of very-low-density lipoprotein particle clearance, regulation of the fatty acid biosynthetic process, regulation of lipoprotein particle clearance, regulation of the fatty acid metabolic process, negative regulation of lipoprotein particle clearance, the lipid metabolic process, protein–lipid complex remodeling, plasma lipoprotein particle remodeling, protein–lipid complex subunit organization, and plasma lipoprotein particle organization. The remaining three GO terms were negative regulation of receptor-mediated endocytosis, organic hydroxyl compound transport, and macromolecular complex remodeling. They were also indirectly associated with lipid metabolism because all of the involved proteins were lipoproteins.

**TABLE 5:** The top 20 most enriched GO terms and involved proteins based on proteomics analysis between PD patients and healthy controls.

Go terms	Category	Involved proteins	*q*-value	Rich factor
Very-low-density lipoprotein particle	CC	O95445, K7ER74, P55056, Q59HB3, P02656, K7ERI9, E1B4S7	0.021	0.292
Triglyceride-rich lipoprotein particle	CC	O95445, K7ER74, P55056, Q59HB3, P02656, K7ERI9, E1B4S7	0.021	0.292
Keratin filament	CC	P04259, P35908, Q04695, Q7RTS7	0.030	0.500
Lipoprotein particle	CC	O95445, K7ER74, P55056, P02656, K7ERI9, Q59HB3, E1B4S7	0.030	0.206
Plasma lipoprotein particle	CC	O95445, K7ER74, P55056, P02656, K7ERI9, Q59HB3, E1B4S7	0.030	0.206
Protein-lipid complex	CC	O95445, K7ER74, P55056, P02656, K7ERI9, Q59HB3, E1B4S7	0.030	0.206
Negative regulation of receptor-mediated endocytosis	BP	K7ER74, P02656, K7ERI9	0.021	1.000
Regulation of very-low-density lipoprotein particle clearance	BP	K7ER74, P02656, K7ERI9	0.021	1.000
Negative regulation of very-low-density lipoprotein particle clearance	BP	K7ER74, P02656, K7ERI9	0.021	1.000
Regulation of fatty acid biosynthetic process	BP	K7ER74, P02656, K7ERI9	0.030	0.750
Regulation of lipoprotein particle clearance	BP	K7ER74, P02656, K7ERI9	0.030	0.750
Regulation of fatty acid metabolic process	BP	K7ER74, P02656, K7ERI9	0.030	0.750
Negative regulation of lipoprotein particle clearance	BP	K7ER74, P02656, K7ERI9	0.030	0.750
Organic hydroxy compound transport	BP	O95445, K7ER74, G5E968, Q59HB3, P02656, K7ERI9, E1B4S7	0.030	0.212
Lipid metabolic process	BP	O95445, Q1W658, O75339, K7ER74, P55056, P02656, K7ERI9, Q59HB3, D6RF20, E1B4S7, P80108	0.030	0.133
Protein-lipid complex remodeling	BP	O95445, K7ER74, P02656, K7ERI9, Q59HB3, E1B4S7	0.030	0.240
Plasma lipoprotein particle remodeling	BP	O95445, K7ER74, P02656, K7ERI9, Q59HB3, E1B4S7	0.030	0.240
Protein-lipid complex subunit organization	BP	O95445, K7ER74, P02656, K7ERI9, Q59HB3, E1B4S7	0.030	0.240
Plasma lipoprotein particle organization	BP	O95445, K7ER74, P02656, K7ERI9, Q59HB3, E1B4S7	0.030	0.240
Macromolecular complex remodeling	BP	O95445, K7ER74, P02656, K7ERI9, Q59HB3, E1B4S7	0.030	0.240

*GO, gene ontology; PD, Parkinson’s disease; CC, cellular component; BP, biological process.*

These differentially expressed proteins were also mapped to KEGG pathways and enriched in five pathways with *p*-values less than 0.05 (Table 6), including ovarian steroidogenesis, the GnRH signaling pathway, neuroactive ligand–receptor interaction, glycosylphosphatidylinositol (GPI)-anchor biosynthesis, and regulation of lipolysis in adipocytes. However, no significant differences can be observed after being adjusted by Benjamini-Hochberg correction (*q*-value > 0.05), indicating that these results were unstable.

**TABLE 6:** Enriched KEGG pathways and involved proteins based on proteomics analysis between PD patients and healthy control.

Enriched KEGG pathway	Involved protein	*p*-value	*q*-value	Rich factor
Ovarian steroidogenesis	Q1W658 C0KRQ8	0.005	0.134	0.667
GnRH signaling pathway	Q1W658 C0KRQ8	0.025	0.217	0.333
Neuroactive ligand-receptor interaction	Q1W658 C0KRQ8	0.034	0.217	0.286
Glycosylphosphatidylinositol (GPI)-anchor biosynthesis	P80108	0.043	0.217	1
Regulation of lipolysis in adipocytes	C0KRQ8	0.043	0.217	1

*KEGG, Kyoto Encyclopedia of Genes and Genomes; PD, Parkinson’s disease.*

## Discussion

Systemic biology is the most useful way to overview the metabolic alterations of an organism in a pathological condition. Here, we have adopted an integrated metabolomics and proteomics analysis of plasma in PD patients to explore its potential pathogenesis for the first time. No significant differences were found in any of the clinical data between these two groups, suggesting the following omics analyses were reasonable.

Researchers from the United States performed the first metabolomics profiling of plasma in PD patients (Bogdanov et al., 2008). They identified altered levels of 8-hydroxy-2-deoxyguanosine, uric acid, and glutathione as potential blood biomarkers for PD patients without further pathway analysis. Other researchers also performed metabolomics analysis of cerebrospinal fluid (Lewitt et al., 2013) and urine (Luan et al., 2015) from PD patients, suggesting various perturbed metabolic pathways related to lipids, energy, fatty acids, bile acids, polyamine, and amino acids (Shao and Le, 2019). In this untargeted metabolomics analysis, significant metabolic differences were found between PD patients and healthy controls from the PLS-DA plot. Eighty-three differentially expressed metabolites were identified between the two groups, most of which were lipid and lipid-like molecules. The lipid and lipid-like molecules were further classified, and sphingolipids accounted for 25%. Furthermore, pathway analysis indicated that sphingolipid metabolism was the only significantly enriched metabolic pathway, and the pathway tended to be activated. Sphingolipids are a class of lipids containing a backbone of sphingoid bases and a set of aliphatic amino alcohols (e.g., ceramides, sphingomyelins, gangliosides, and cerebrosides). The involvement of sphingolipid metabolism was first reported in a yeast model of Parkinson’s disease (Lee et al., 2011). The *Saccharomyces cerevisiae* lipid elongase null mutants exhibited severe growth defects, accumulation of reactive oxygen species, aberrant protein trafficking, and a dramatic decrease in the survival of aged cells. α-Synuclein (α-syn) is a small soluble synaptic protein that is the major proteinaceous component of Lewy bodies, the pathological hallmark of Parkinson’s disease (Jo et al., 2004). It was reported that the toxicity of α-syn could be enhanced as a result of the disruption of ceramide-sphingolipid homeostasis in the endoplasmic reticulum (Lee et al., 2011). Whereas Gaucher disease-related sphingolipids (glucosylceramide, glucosylsphingosine, sphingosine, sphingosine-1-phosphate) are reported to induce α-syn aggregation, glucosylsphingosine can trigger the formation of oligomeric α-syn in human neurons (Taguchi et al., 2017). Autophagy can be impaired by the increased levels of glucosylceramide and sphingomyelin while also enhanced by the increased levels of ceramide (Indellicato and Trinchera, 2019). In total, the disruption of the sphingolipid metabolism leads to α-syn accumulation, influences the traffic of vesicles toward lysosomes, and affects autophagy. Mutations of glucocerebrosidase 1, an enzyme hydrolyzing glucocerebroside (a subclass of sphingolipids), are the most common genetic risk factor for PD, although the underlying biological processes are poorly understood (Gan-Or et al., 2008). Metabolomics analysis mainly focuses on small molecules (<1 KD), and in this research a proteomics analysis was further performed.

A lot of proteomics research has been conducted in PD patients, mainly from brain tissues (substantia nigra, thalamus, locus coeruleus, olfactory bulb, and cerebral cortex), biofluids (cerebrospinal fluid, tears, and blood) and subcellular structures (mitochondria) (Dixit et al., 2019). Most of this research referred to pathways involved in “mitochondrial dysfunction, oxidative stress, protein aggregation or degradation, autophagy, and inflammation”(Ren et al., 2015; Monti et al., 2018). Here, forty proteins were differentially expressed in PD patients from the plasma proteomics analysis and the levels of all the seven apolipoproteins (apolipoprotein C-I, apolipoprotein C-III, protein APOC4-APOC2, apolipoprotein C-IV, apolipoprotein B variant, apolipoprotein B, and apolipoprotein M) were significantly decreased. Another former study reported decreases of apolipoprotein AII and apolipoprotein E in Parkinson’s disease (Zhang et al., 2008). The decrease of apolipoprotein B-100 (Lehnert et al., 2012) and apolipoprotein A-I (Zhang et al., 2012) was also reported in some studies. There are a large number of lipids in the central nervous system, and about one-fourth of the total cholesterol has a function or is stored in the brain. These apolipoproteins can influence the deposition process of many proteins in neurodegenerative diseases, such as α-syn in PD (Zhang et al., 2012). According to the enriched GO analysis, five of the six top ranking GO terms from cellular components were directly associated with lipids, and eleven of the fourteen top ranking GO terms from biological processes were directly associated with lipid metabolism. Keratin filament from the cellular component is a part of the intermediate filament and constitutes the cytoskeleton while Lewy bodies are the accumulation of neurofilaments (intermediate filament) in Parkinson’s disease. The underlying pathogenesis needs further research to confirm. The remaining three GO terms from biological processes were also related with lipid metabolism, as all the involved proteins were lipoproteins. KEGG pathway analysis was performed with those differentially expressed proteins and it seemed to be enriched in five pathways. The GnRH signaling pathway and ovarian steroidogenesis directly regulate steroid hormones and participate in the production of steroids. These processes belong to neuroactive ligand–receptor interaction. Glycosylphosphatidylinositol (GPI)–anchor biosynthesis and regulation of lipolysis in adipocytes are direct detailed processes of lipid metabolism. However, the results were unstable after Benjamini-Hochberg correction, as no significant differences could be observed (*q*-value > 0.05).

Both sphingolipids and apolipoproteins participate in plasma lipid metabolism, and are mainly related with atherosclerotic diseases. A number of lipidomics studies have reported specific lipid alterations in the brain or plasma from PD patients, such as the composition alteration of lipid rafts in the frontal cortex (Alecu and Bennett, 2019). The interaction of α-syn and the synaptic membrane is thought to be critical in Parkinson’s disease and has been explored for many years (Kubo et al., 2005). It is reported that lipids and the ratio of lipids to proteins can regulate the aggregation propensity of α-syn (Galvagnion, 2017). α-Syn can interact with the lipid membrane, and that interaction further affects the oligomerization and aggregation of α-syn (Suzuki et al., 2018). The alteration of lipid chemical properties can also induce α-syn aggregation and affect the balance between functional and aberrant behavior of α-syn (Galvagnion et al., 2016). In addition, α-syn can bind to specific lipid molecules, and these lipid–protein conjugates can further help the transport of α-syn in the blood or across the blood-brain barrier (Emamzadeh and Allsop, 2017), enhance its interaction with synaptic membranes, and interfere with the catalytic activity of cytoplasmic and lysosomal lipases, thereby disrupting lipid metabolism (Alecu and Bennett, 2019). In the meantime, lipid dysregulation can also promote PD pathogenesis through oxidative stress and inflammation reaction. Apolipoprotein M can increase the level of circulating sphingosine 1-phosphate, activate the following signaling pathway, and induce inflammation reaction (Kurano et al., 2013). Platelet activating factors, a lipid proinflammatory mediator, play an important role in modulating progressive neurodegeneration in PD patients by intracellular trafficking of α-syn. Furthermore, there are a large number of genetic risk factors of PD involved in lipid metabolism, including *PLA2G6* and *SCARB2*. These two genes are directly or indirectly involved in glycerophospholipid and sphingolipid metabolism (Alecu and Bennett, 2019). Mutations in the glucocerebrosidase 1 gene, which encodes a degrading enzyme for the glycolipid glucosylceramide, are regarded as strong risk factors for PD and dementia with Lewy bodies, and glucosylceramide has been confirmed to promote toxic conversion of α-syn. Moreover, pathological research has demonstrated the existence of α-syn in the brains of patients with lysosomal storage disorders, in which glycosphingolipids is found to be accumulated (Suzuki et al., 2018). In addition, α-syn can antagonize neurotrophic signaling of TrkB by repressing TrkB lipid raft distribution, decreasing its internalization, and reducing its axonal trafficking (Kang et al., 2017). The possible pathogenesis is that α-syn sequesters the early peroxidation products of fatty acids, thereby reducing the level of highly reactive lipid species (De Franceschi et al., 2017). All in all, dysregulated lipid metabolism is implicated in Parkinson’s disease, which might open a new therapeutic method for modifying Parkinson’s disease.

## Conclusion

Integrated proteomics and metabolomics analysis reveals that Parkinson’s disease is associated with plasma lipid metabolic disturbance. The activated sphingolipid metabolism and decreased apolipoproteins are the probable potential pathogenesis for PD.

## Data Availability Statement

The datasets presented in this study can be found in the supplementary materials (Supplementary Table S1) and online repository PeptideAtlas, with accession number PASS01568 (http://www.peptideatlas.org/PASS/PASS01568).

## Ethics Statement

The studies involving human participants were reviewed and approved by the Ethics committee of the First Affiliated Hospital of Chongqing Medical University. The patients/participants provided their written informed consent to participate in this study.

## Author Contributions

LH and Y-DW designed this study. LH, C-QL, QQ, C-CZ, and M-XD assessed the clinical data. LH, X-MX, and YL performed the experiments. M-XD, Y-LH, and G-HC wrote and revised the manuscript.

## Conflict of Interest

The authors declare that the research was conducted in the absence of any commercial or financial relationships that could be construed as a potential conflict of interest.

## Footnotes

1 https://metlin.scripps.edu

2 http://www.hmdb.ca

3 http://www.metaboanalyst.ca

## Supplementary Material

TABLE S1 The matrix of metabolomics data included in the paper.
